# Another Case of Maternal Impressions, with Singular Malformations

**Published:** 1881-06

**Authors:** 


### ANOTHER CASE OF MATERNAL IMPRESSIONS, WITH SINGULAR MALFORMATIONS.

I was called January 19th, to attend a young unmarried woman in premature confinement. The patient supposed herself about five months advanced, which I presume was not far from correct. The labor was tedious and proved to be a breech presentation. After the birth of the body I had more than usual difficulty in the extraction of the head, especially in view of the age of the fœtus. After the birth was completed, however, upon examination, I found the hair on the head very abundant, and extended down evenly and uniformly over the whole forehead, a compact matted mass continuous with the eyebrows. This peculiar development of hair gave the head a very singular expression—very closely resembling a squirrel. She made a very satisfactory explanation by claiming that when she was only a few weeks gone in pregnancy she had been greatly startled by a pet squirrel that had attempted to bite, her!

Upon further examination I found two teeth in the lower jaw, the two lower incisors, quite well developed, and showing outside of the gum. This was also explained promptly by her frequent repugnance at the sight of some poorly constructed false teeth, worn by her aunt!

In this matter of teeth at birth, as the occurrence is comparatively rare, I have clipped this note from the January number of the *North Carolina Medical Journal*:

We are indebted to Dr. Wm. J. Love for the following notes of unusual triplets, born in Wilmington in December:

Mrs. Black gave birth to triplets on the 11th, between Chestnut and Mulberry streets. This was her second confinement and second pregnancy. She is a North Carolinian, aged 45 years.

The presentations were all of the head, the labor was natural. Two of the children were girls, one a boy.

The first girl weighed 4¼ lbs. She had *two middle upper incisors* and *two upper canines*, and lived five hours.

The second girl weighed five pounds. She had *two middle upper incisors* and *left upper canine*, and lived five hours.

The boy weighed 6¼ pounds. He had *four upper incisors*, and *two upper canines* nearly through. He lived five hours.

